# Supplementary material for: Multi-target genome editing reduces polyphenol oxidase activity in wheat (Triticum aestivum L.) grains
Source: Front Plant Sci. 2023 Sep 15;14:1247680. doi: 10.3389/fpls.2023.1247680 (PMC10541959; doi:10.3389/fpls.2023.1247680)
Supplement: Supplementary file 1 [file DataSheet_1.pdf]

Supplementary Material

Multi-target genome editing reduces polyphenol oxidase activity in wheat (*Triticum aestivum* L.) grains

Forrest Wold-McGimsey[1], Caitlynd Krosch[1], Rocío Alarcón Reverte[1,2], Karl Ravet[1], Andrew Katz[1], John Stromberger[1], Richard Esten Mason[1] and Stephen Pearce[1,2]*.

*** Correspondence:**
Stephen Pearce.
Stephen.pearce@rothamsted.ac.uk

This document includes Tables S1 to S13 and Figure S1:

Table S1: Presence/absence variation in *PPO1* and *PPO2* genes.

Table S2: *PPO* genes in 'Chinese Spring' and 'Fielder'.

Table S3: Alleles of *PPO1* and *PPO2* genes in 'Fielder'.

Table S4: Details of three sgRNAs designed to target *PPO1* and *PPO2* genes in wheat.

Table S5: Predicted off-target effects of the selected sgRNA used in this study.

Table S6: PPO activity in wild-type, T_1 and T_2 lines from the varieties 'Fielder', 'Guardian' and 'Steamboat'.

Table S7: Editing events in seven *PPO1* and *PPO2* genes in selected T_1 and T_2 'Fielder' individuals.

Table S8: Editing events in seven *PPO1* and *PPO2* genes in selected T_1 and T_2 'Guardian' individuals.

Table S9: Editing events in seven *PPO1* and *PPO2* genes in selected T_1 and T_2 'Steamboat' individuals.

Table S10: Alleles of *PPO1* and *PPO2* genes in ‘Kronos’.

Table S11: Mean brightness values of dough samples.

Table S12: Mismatches between the sgRNA described by Zhang *et al*. 2021 and the seven *PPO1* and *PPO2* genes in the ‘Fielder’ genome.

Table S13: Mismatches between the sgRNA used in the current study and orthologous *PPO1* and *PPO2* genes from closely related species.

Table S14: Primers used for PCR in the current study.

Figure S1: Position of *PPO1* and *PPO2* genes on chromosome 2B in the wheat landrace ‘Chinese Spring’ and the variety ‘CDC Landmark’.

Table S1: Presence/absence variation in *PPO1* and *PPO2* genes in 17 common wheat varieties.

Variety	*PPO1-A1*	*PPO2-A1*	*PPO1-B1*	*PPO1-B2*	*PPO2-B1*	*PPO1-D1*	*PPO2-D1*
Arina	Present	Present	Present	Present	Present	Present	Present
Chinese Spring	Present	Present	Present	Present	Present	Present	Present
Claire	Present	Present	Present	Present	Present	Present	Present
Fielder	Present	Present	Present	Present	Present	Present	Present
Jagger	Present	Present	Present	Present	Present	Present	Present
Norin 61	Present	Present	Present	Present	Present	Present	Present
Robigus	Present	Present	Present	Present	Present	Present	Present
Lancer	Present	Present	Present	Present	Absent	Present	Present
Julius	Present	Present	Present	Present	Absent	Present	Present
Cadenza	Present	Present	Absent	Absent	Present	Present	Present
Landmark	Present	Present	Absent	Absent	Present	Present	Present
Mace	Present	Present	Absent	Absent	Present	Present	Present
Paragon	Present	Present	Absent	Absent	Present	Present	Present
Stanley	Present	Present	Absent	Absent	Present	Present	Present
SY Mattis	Present	Present	Absent	Absent	Present	Present	Present
Guardian	Present	Present	Absent	Absent	Present	Present	Present
Steamboat	Present	Present	Absent	Absent	Present	Present	Present

Table S2: *PPO* genes in 'Chinese Spring' and 'Fielder'. Gene names are presented according to the guidelines of the Wheat Initiative (Boden et al., 2023) and consistent with previously described allelic variation. Previously proposed gene names are listed (Liu et al., 2020), alongside gene IDs from Chinese Spring (IWGSC v1.2) and Fielder (v1) genome assemblies. Genes that are absent from the 'Chinese Spring' genome assembly are marked with a dash (-). The seven target *PPO1* and *PPO2* genes are separated from non-target *PPO* genes by a horizontal line.

Gene name	Previous gene name (Liu et al. 2020)	Chinese Spring	Fielder
PPO1-A1	*PPO2A-1*	*TraesCS2A02G468200*	*TraesFLD2A01G514000*
PPO1-B1	*PPO2B-1*	*TraesCS2B02G491000*	*TraesFLD2B01G529900*
PPO1-B2	*PPO2B-2*	*TraesCS2B02G491100*	*TraesFLD2B01G530000*
PPO1-D1	*PPO2D-1*	*TraesCS2D02G468200*	*TraesFLD2D01G517900*
PPO2-A1	*PPO2A-2*	*TraesCS2A02G468500*	*TraesFLD2A01G514300*
PPO2-B1	*PPO2B-3*	*TraesCS2B02G491400*	*TraesFLD2B01G530300*
PPO2-D1	*PPO2D-2*	*TraesCS2D02G468600*	*TraesFLD2D01G518200*
PPO3-A1	*PPO3A-1*	*TraesCS3A02G326600*	*TraesFLD3A01G347800*
PPO3-A2	-	-	*TraesFLD3A01G347700*
PPO3-B1	*PPO3B-1*	*TraesCS3B02G355900*	*TraesFLD3B01G404300*
PPO3-B2	*PPO3B-2*	*TraesCS3B02G568600*	*TraesFLD3B01G637100*
PPO4-A1	*PPO5A-1*	*TraesCS5A02G549200*	*TraesFLD5A01G586800*
PPO4-B1	*PPO4B-1*	*TraesCS4B02G385600*	*TraesFLD4B01G423700*
PPO4-D1	*PPO4D-1*	*TraesCS4D02G358500*	*TraesFLD4D01G404500*
PPO4-D2	*PPO4D-2*	*TraesCS4D02G359200*	*TraesFLD4D01G405400*
PPO5-A1	*PPO6A-1*	*TraesCS6A02G041900*	*TraesFLD6A01G054300*
PPO5-B1	*PPO6B-2*	*TraesCS6B02G056900*	*TraesFLD6B01G067500*
PPO5-D1	*PPO6D-1*	*TraesCS6D02G048400*	*TraesFLD6D01G053200*
PPO6-B1	*PPO6B-1*	*TraesCS6B02G016400*	*TraesFLD6B01G018100*
PPO6-B2	-	-	*TraesFLD6B01G017900*
PPO6-B3	-	-	*TraesFLD6B01G018000*
PPO6-B4	-	-	*TraesFLD6B01G018200*
PPO6-B5	-	-	*TraesFLD6B01G017300*
PPO7-A1	*PPO7A-1*	*TraesCS7A02G054500*	*TraesFLD7A01G056900*
PPO7-D1	*PPO7D-1*	*TraesCS7D02G049700*	*TraesFLD7D01G064600*

Table S3: Alleles of *PPO1* and *PPO2* genes in 'Fielder'. Wild-type alleles are predicted to encode full-length, functional PPO proteins with all conserved domains.

Gene name	Fielder Gene ID	Polymorphism	Effect
PPO1-A1	*TraesFLD2A01G514000*	54-bp deletion in third exon (1662_1713del)	Frame shift, stop codon introduced at amino acid 423.
PPO1-B1	*TraesFLD2B01G529900*	Wild-type	
PPO1-B2	*TraesFLD2B01G530000*	1-bp deletion in third exon (1977delC)	Frame shift, stop codon introduced at amino acid 525.
PPO1-D1	*TraesFLD2D01G517900*	Wild-type	
PPO2-A1	*TraesFLD2A01G514300*	Wild-type	
PPO2-B1	*TraesFLD2B01G530300*	Wild-type	
PPO2-D1	*TraesFLD2D01G518200*	1-bp substitution in first exon (20C>A)	Stop codon introduced at amino acid 7.

Table S4: Details of three sgRNAs designed to target *PPO1* and *PPO2* genes in wheat. Quality scores include: RS2 - Rule Set 2 quality score derived from the algorithm described by (Doench et al., 2016). Overall score - weighted average of RS2 and cutting frequency determination from the wheatCRISPR tool (Cram et al., 2019). CRISPRP score - quality score determined by the CRISPR-P 2.0 tool (Liu et al., 2017). The total number of T_0 plants represents the number of independently-derived plants confirmed by PCR to carry the transgene. A T_0 plant was considered "edited" when induced edits in at least one target *PPO1* or *PPO2* gene were detected by Sanger sequencing. All results described in this manuscript used the first sgRNA in this table.

							**T_0 plants**	
**Position (IWGSC v1.2)**	**Strand**	**Sequence**	**PAM**	**Overall score**	**RS2**	**CRISPRp score**	**Total**	**Edited**
chr2A: 712,188,023	Antisense	CGTGGTGCGCGAAGAAGATG	GGG	0.58	0.63	0.44	10	10
chr2A: 712,188,158	Antisense	GGTCCGGCTGGTCGCCGGCG	CGG	0.57	0.60	0.30	4	0
chr2A: 712,188,155	Sense	GCCGGCGACCAGCCGGACCC	GGG	0.51	0.34	0.06	11	0

Table S5: Predicted off-target effects of the selected sgRNA used in this study (CGTGGTGCGCGAAGAAGATG). Cutting Frequency Determination (CFD) represents the predicted cutting efficiency of an off-target sequence relative to the on-target sequence, ranging from 0 (no predicted activity), to 1.0 (full predicted activity). The first row represents the sgRNA selected sequence targeting all seven *PPO1* and *PPO2* genes (CFD = 1). The gene IDs of other genes targeted in the promoter are listed. Off-target effects were predicted using the wheatCRISPR tool (Cram et al., 2019).

Protospacer sequence	PAM	CFD	Mismatches	Coding region	Promoter	Other genic region	Intergenic	Targeted gene
CGTGGTGCGCGAAGAAGATG	GG	1	0	7	0	0	0	*TraesCS2A02G468200*<br>*TraesCS2A02G468500*<br>*TraesCS2B02G491000*<br>*TraesCS2B02G491100*<br>*TraesCS2B02G491400*<br>*TraesCS2D02G468200*<br>*TraesCS2D02G468600*
TGTGGCACGCGAAGAAGATG	GG	0.91	3	0	1	0	0	*TraesCS7B02G293100*
TGTGGCACGTGAAGAAGATG	GG	0.86	4	0	1	0	0	*TraesCS4B02G141200*
CATGGCACGTGAAGAAGATG	GG	0.72	4	0	2	2	0	*TraesCS4A02G553800LC*<br>*TraesCS7B02G226300*
CGTGAAGCACGAAGAAGATG	GG	0.48	3	0	0	0	1	
CGGGGCACGCGAAGAAGATG	GG	0.46	3	0	0	0	1	
CGTCGTGTGCAAAGAAAATG	GG	0.43	4	0	1	0	0	*TraesCS3D02G054900*
TGAGGTCTGCGAAGAAGATG	GG	0.43	4	0	1	0	0	*TraesCS7A02G282200*
CCTAGTCCGCGAAGAAAATG	GG	0.4	4	0	1	0	0	*TraesCS1A02G195500*
GCTGGAGCGCGAAGAAGATT	GG	0.38	4	0	1	0	0	*TraesCS6B02G572000LC*

Table S6: PPO activity in wild-type, T_1 and T_2 lines from the varieties 'Fielder', 'Guardian' and 'Steamboat'. Mean PPO activity, the number of biological replicates (n), standard deviation and two-tailed Student's T-test result showing the difference with each corresponding wild-type line are shown.

Genotype	Line	Generation	Mean PPO activity (A475)	% of wild-type	n	Standard deviation	*P* value
Ripper	Wild-type	Wild-type	0.797	-	10	0.019	
Platte	Wild-type	Wild-type	0.177	-	10	0.008	
Kronos	Wild-type	Wild-type	0.050	-	10	0.007	
Fielder	Wild-type	Wild-type	0.132	-	16	0.064	
	81.5a	T_1	0.073	54.9	12	0.040	0.0058
	81.7a	T_1	0.035	26.7	16	0.033	2.14 e^{-5}
	81.8b	T_1	0.117	88.5	15	0.048	0.46
	81.12a	T_1	0.026	19.3	16	0.022	5.86 e^{-6}
	81.15b	T_1	0.044	33.3	16	0.027	6.12 e^{-6}
	81.16a	T_1	0.161	121.7	15	0.044	0.16
	81.19a	T_1	0.070	52.8	16	0.030	0.0021
	81.5a.1	T_2	0.025	19.1	17	0.016	6.42 e^{-6}
	81.5a.6	T_2	0.018	13.8	17	0.017	2.91 e^{-6}
Guardian	Wild-type	Wild-type	0.263	-	10	0.052	
	19.1a	T_1	0.023	8.7	17	0.011	1.00 e^{-7}
	19.2a	T_1	0.022	8.4	18	0.020	3.91 e^{-8}
	19.1a.6	T_2	0.014	5.1	16	0.018	3.90 e^{-8}
	19.2a.1	T_2	0.029	11.1	16	0.018	6.20 e^{-8}
Steamboat	Wild-type	Wild-type	0.163	-	10	0.053	
	23.2a	T_1	0.032	19.6	15	0.020	1.34 e^{-5}
	23.2a.8	T_2	0.012	7.6	16	0.011	1.73 e^{-5}

Table S7: Editing events in seven *PPO1* and *PPO2* genes in selected T_1 and T_2 'Fielder' individuals. Where mutations are heterozygous, both allele types are described. WT = wild-type allele. * = natural allele is expected to encode a non-functional protein in Fielder (Table S3).

**T_1**	**81.5a.1**	**81.5a.6**
*PPO1-A1**	[1464_1465delTC]/[1465delC]	[1464_1465delTC]
PPO2-A1	[1274_1275insC]/[1273_1274delTC]	[1274_1275insC]
PPO1-B1	[1512_1518delATCTTC]	[1512_1518delATCTTC]
*PPO1-B2**	[1369_1383delTTCTTCGCGCACCAC]	[1369_1383delTTCTTCGCGCACCAC]
PPO2-B1	[1295_1296insT]	[1295_1296insT]
PPO1-D1	[1273_1274delTC]	[1270subA;1274delC]
*PPO2-D1**	No edits	[1266_1267insG;1266_1279delTTCTTCGCGCACC]
**T_2**	**81.5a.1.6; 81.5a.1.5; 81.5a.1.8.**	**81.5a.6.1; 81.5a.6.4; 81.5a.6.7; 81.5a.6.13.**
*PPO1-A1**	[1465delC]	[1464_1465delTC]
PPO2-A1	[1273_1274delTC]	[1274_1275insC]
PPO1-B1	[1512_1518delATCTTC]	[1512_1518delATCTTC]
*PPO1-B2**	[1369_1383delTTCTTCGCGCACCAC]	[1369_1383delTTCTTCGCGCACCAC]
PPO2-B1	[1295_1296insT]	[1295_1296insT]
PPO1-D1	[1273_1274delTC]	[1270subA;1274delC]
*PPO2-D1**	No edits	[1266_1267insG;1266_1279delTTCTTCGCGCACC]

T_2 line 81.5a.1.11 has the same genotype, except biallelic alleles in *PPO1-A1* and *PPO1-A2*.

Table S8: Editing events in five *PPO1* and *PPO2* genes in selected T_1 and T_2 'Guardian' individuals. Where mutations are heterozygous, both allele types are described, separated by "/".

**T_1**	**19.1a.6**	**19.2a.1**
PPO2A-1	[1299_1300insA]/[1296_1304delCCATCTTCT]	[1299_1300insA]/[1299_1300insT]
PPO2A-2	[1230delC]/[1232subT]	[1232_1235delTCTT]/[1230_1234delCTTCT]
PPO2B-3	[1367_1382delTCTTCTTCGCGCACCA]	[1367_1368insT]
PPO2D-1	[1341_1342delTC]	[1341_1343delTCT]/[1341_1342insC]
PPO2D-2	[1263_1270delCATCTTCT]	[1266_1271delTCTTCT]/ [1235_1271delCGCGCGACCCCATCTTCTT]
**T_2**	**19.1a.6.5**	**19.2a.1.3**
PPO2A-1	[1299_1300insA]/[1296_1304delCCATCTTCT]	[1299_1300insA]
PPO2A-2	[1230delC]/[1232subT]	[1230_1234delCTTCT]
PPO2B-3	[1367_1382delTCTTCTTCGCGCACCA]	[1367_1368insT]
PPO2D-1	[1341_1342delTC]	[1341_1342insC]
PPO2D-2	[1263_1270delCATCTTCT]	[1266_1271delTCTTCT]

Table S9: Editing events in five *PPO1* and *PPO2* genes in selected T_1 and T_2 ‘Steamboat’ individuals. Where mutations are heterozygous, both allele types are described, separated by “/”.

**T_1**	**23.2a.8**
PPO2A-1	[1273_1299delCTTCTTCTCGGCGGCGCGCGACCCCAT]
PPO2A-2	[1229_1230insA]
PPO2B-3	[1368delC]/[1368_1369insC]
PPO2D-1	[1342delC]
PPO2D-2	[1267_1269delTTC]
**T_2**	**23.2a.8.15**
PPO2A-1	[1273_1299delTCTTCTCGGCGGCGCGCGACCCCATCT]
PPO2A-2	[1229_1230insA]
PPO2B-3	[1368delC]
PPO2D-1	[1342delC]
PPO2D-2	[1267_1269delTTC]

Table S10: Alleles of *PPO1* and *PPO2* genes in ‘Kronos’. *PPO* gene sequences were used as queries in BLAST searches against the Kronos Elv1.1 genome assembly.

Gene name	Fielder Gene ID	Polymorphism	Effect
PPO1-A1	*TraesFLD2A01G514000*	Wild-type	
PPO2-A1	*TraesFLD2A01G514300*	Wild-type	
PPO1-B1	*TraesFLD2B01G529900*	Deleted	
PPO1-B2	*TraesFLD2B01G530000*	Deleted	
PPO2-B1	*TraesFLD2B01G530300*	1 nucleotide substitution in first exon, (20C>A)	Stop codon introduced at amino acid 7.

Table S11: Mean brightness values of dough samples measured at seven time points (n = 5). * $P < 0.05$, ** $P < 0.01$.

Genotype	Line	Mean brightness (L*)						
		0 h	30 min	1 h	2 h	4 h	8 h	24 h
**Fielder**	WT	65.318	63.166	62.414	61.186	59.458	56.736	48.508
	81.5a.1	68.248**	65.388**	64.704**	63.806**	62.622**	61.022**	56.866**
	81.5a.6	68.622**	65.666**	64.974**	64.108**	62.842**	61.298**	57.338**
**Steamboat**	WT	62.972	59.184	58.158	56.762	54.816	52.32	46.408
	23.2a.8	63.846	59.876	58.918	57.656	55.972	54.022	51.524**
**Guardian**	WT	61.05	57.062	55.846	54.418	52.48	49.704	42.426
	19.1a.6	62.602*	57.712	56.528	55.096	53.336*	51.196*	47.83**
	19.2a.1	62.968**	58.816**	57.746**	56.45**	54.726**	52.668**	49.806**
**Ripper**	WT	59.954	56.17	55.158	53.736	51.68	48.822	42.638

Table S12: Mismatches between the sgRNA described by Zhang *et al*. (2021) and the seven *PPO1* and *PPO2* genes in the 'Fielder' genome. This sgRNA matches 100% only with *PPO1-A1*, *PPO1-B2* and *PPO1-D1*. The protospacer sequence (5'-GAAGAAGACGCTGCTGTTCC-3') designed to the forward strand is between positions 1,291 and 1,310 bp downstream of the initiating ATG codon of *PPO1-A1* based on the 'Fielder' gene model. Mismatches are highlighted in bold. PAM sequence is in red.

Gene name	Fielder gene ID	sgRNA sequence	Number of mismatches
PPO1-A1	*TraesFLD2A01G514000*	GAAGAAGACGCTGCTGTTCCTGG	0
PPO2-A1	*TraesFLD2A01G514300*	**C**AAGAAGAC**T**C**G**GCTGTTC**A**TGG	4
PPO1-B1	*TraesFLD2B01G529900*	GAAGAAGACG**T**TGCTGTTCCTGG	1
PPO1-B2	*TraesFLD2B01G530000*	GAAGAAGACGCTGCTGTTCCTGG	0
PPO2-B1	*TraesFLD2B01G530300*	**C**AAGAAGAC**T**C**G**GCTGTTC**A**TGG	4
PPO1-D1	*TraesFLD2D01G517900*	GAAGAAGACGCTGCTGTTCCTGG	0
PPO2-D1	*TraesFLD2D01G518200*	**C**AAGAAGAC**T**C**C**GCTGTTC**A**TGG	4

Table S13: Mismatches between the sgRNA used in the current study and orthologous *PPO1* and *PPO2* genes from closely related species. Mismatches are highlighted in bold. PAM sequence is in red. * The mismatch in *ScPPO1* alters the PAM sequence from CCC to CCT, which is still permissible for editing.

Species	Gene name	Gene ID	sgRNA sequence	Number of mismatches
Triticum aestivum	*TaPPO1-A1*	*TraesFLD2A01G514000*	CCCCATCTTCTTCGCGCACCACG	0
Hordeum vulgare	*HvPPO1*	*HORVU.MOREX.r3.2HG0194690*	CCCCATCTTCTTCGCGCACCACG	0
Hordeum vulgare	*HvPPO2*	*HORVU.MOREX.r3.2HG0194630*	CCCCATCTTCTTCGCGCACCACG	0
Secale cereale	*ScPPO1*	*SECCE2Rv1G0121020*	CC**T**CATCTTCTTCGCGCACCACG	1*
Secale cereale	*ScPPO2*	*SECCE2Rv1G0121070*	CCCCATCTTCTTCGCGCACCACG	0
Zea mays	*ZmPPO1*	*Zm00001eb431220*	CCC**GG**T**G**TTCTTCGCGCACCACG	3
Zea mays	*ZmPPO2*	*Zm00001eb428900*	CCC**GC**TCTTCTTCGCGCACCACG	2
Zea mays	*ZmPPO3*	*Zm00001eb171410*	CCC**GC**TCTTCT**A**C**T**CGCACCACG	4
Zea mays	*ZmPPO4*	*Zm00001eb388240*	CCCCATCTTCTTC**C**CGCACCAC**A**	2
Oryza sativa	*OsPPO1*	*Os04g0624500*	CCC**GG**T**G**TTCTTCGCGCACCACG	3
Oryza sativa	*OsPPO2*	*Os04g0624450*	CCC**GG**T**G**TTCTTCGCGCACCACG	3
Sorghum bicolor	*SbPPO1*	*SORBI_3007G068500*	CCC**GC**TCTTCT**A**C**T**CGCACCACG	4
Sorghum bicolor	*SbPPO2*	*SORBI_3006G181300*	**T**CC**AC**TCTTCTTCGCGCACCACG	3
Sorghum bicolor	*SbPPO3*	*SORBI_3006G181400*	**T**CC**GC**TCTTCTTCGCGCACCACG	3
Sorghum bicolor	*SbPPO4*	*SORBI_3010G192700*	CCCCATCTTCT**A**C**C**CGCACCACG	2

Table S14: Primers used for PCR in the current study. To amplify the *PPO1-D1* and *PPO2-D1* genes, different PCR assays were used in different genotypes due to sequence variation.

Target	Primer Name	Primer Sequence (5’-3’)	Annealing temp (°C)	Annealing time (seconds)	Amplicon size (bp)
PPO1-A1	PPO-A1_F5	AAAATATTGTTAACATAACCACAGAGTT	57	60	972
	PPO-A1_R5	CCAGAGCGGAGCACATTAC			
PPO2-A1	PPO-A2_F2	GTAACGTACACCACATAATAAATACGC	57	60	1,200
	PPO-A2_R2	GCCTCCTCCTTCTCCTTGTC			
PPO1-B1	PPO-B1_F3	GACGCCAATATCCCAAGAGA	61	30	933
	PPO-B1_R3	ACCCTGGGCCTCGTCACA			
PPO1-B2	PPO-B2c_F	GACGCGGTACCAAAATCTCTC	59	30	493
	PPO-B2_R	TCCTGCGGGATCTCTTGC			
PPO2-B1	PPO-B1_F3	GACGCCAATATCCCAAGAGA	60	30	725
	PPO-B3_R2	GGCCTCGTCACCGTCAT			
PPO1-D1 (Steamboat)	PPO-D1a_F	CCTCAAGAATCCCTAAACAAAATGAG	59	30	1,008
	PPO-D1a_R	GACGCGAGAGCGGACCACATTAG			
PPO1-D1 (Guardian, Fielder)	PPO-D1bc_F2	CGGTCATCTACGCCAACAGA	61	30	627
	PPO-D1bc_R2	CCTCGTCGTAGAAGAGGAAGC			
PPO2-D1 (Steamboat)	PPO-D2a_F	TGTCGGGTGCCAAGAAGACTA	65	30	275
	PPO-D2_R	CCAGTCGGCGTCGGC			
PPO2-D1 (Guardian, Fielder)	PPO-D2b_F	TGTCGAGTGCCAAGAAGACTCC	65	30	275
	PPO-D2_R	CCAGTCGGCGTCGGC			
JD633 plasmid backbone	TaU6-promoter_F	TAGGAGGGAATCGAACTAGG	57	60	400
	gRNA-scaffold_R	CTTTTTCAAGTTGATAACGG			
Hygromycin resistance gene	HYG_F	CTATTTCTTTGCCCTCGGACGAGTGC	55	60	1,026
	HYG_R	ATGAAAAAGCCTGAACTCACCGCGAC			
TaCas9	TaCDP-1_F	GGCAGCCCGGAGGACAACGAGC	60	60	350
	TaCDP-1_R	GAGAGGTCGATCCTCGTCTCGTA			

Figure S1: Position of *PPO1* and *PPO2* genes on chromosome 2B in the wheat landrace 'Chinese Spring' and the variety 'CDC Landmark'. Gene positions are drawn to scale and homologous genes linked by lines determined using the Triticeae Gene Tribe microhomology tool (Chen et al., 2020).

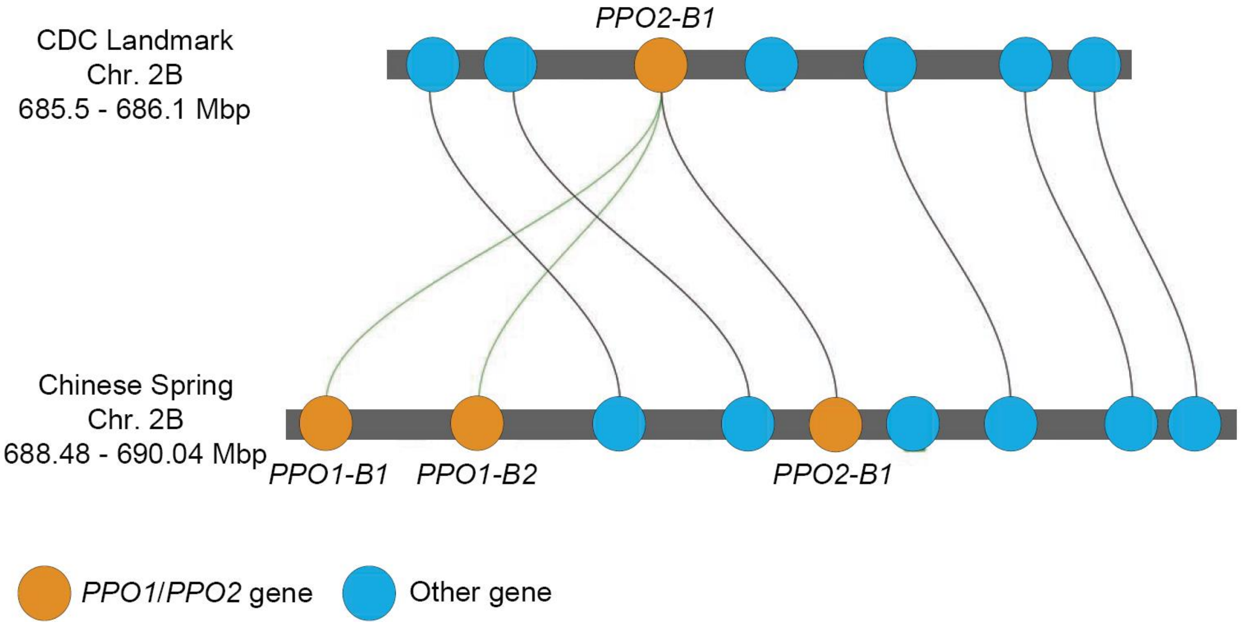

REFERENCES

Boden, S.A., McIntosh, R.A., Uauy, C., Krattinger, S.G., Dubcovsky, J., Rogers, W.J., Xia, X.C., Badaeva, E.D., Bentley, A.R., Brown-Guedira, G., Caccamo, M., Cattivelli, L., Chhuneja, P., Cockram, J., Contreras-Moreira, B., Dreisigacker, S., Edwards, D., González, F.G., Guzmán, C., Ikeda, T.M., Karsai, I., Nasuda, S., Pozniak, C., Prins, R., Sen, T.Z., Silva, P., Simkova, H. and Zhang, Y. (2023) Updated guidelines for gene nomenclature in wheat. *Theor Appl Genet* **136**, 72.

Chen, Y., Song, W., Xie, X., Wang, Z., Guan, P., Peng, H., Jiao, Y., Ni, Z., Sun, Q. and Guo, W. (2020) A collinearity-incorporating homology inference strategy for connecting emerging assemblies in the Triticeae tribe as a pilot practice in the plant pangenomic era. *Mol Plant* **13**, 1694-1708.

Cram, D., Kulkarni, M., Buchwaldt, M., Rajagopalan, N., Bhowmik, P., Rozwadowski, K., Parkin, I.A.P., Sharpe, A.G. and Kagale, S. (2019) WheatCRISPR: a web-based guide RNA design tool for CRISPR/Cas9-mediated genome editing in wheat. *BMC Plant Biol* **19**, 474.

Doench, J.G., Fusi, N., Sullender, M., Hegde, M., Vaimberg, E.W., Donovan, K.F., Smith, I., Tothova, Z., Wilen, C., Orchard, R., Virgin, H.W., Listgarten, J. and Root, D.E. (2016) Optimized sgRNA design to maximize activity and minimize off-target effects of CRISPR-Cas9. *Nat Biotechnol* **34**, 184-191.

Liu, C., Zhou, Z., Wu, Z., Qin, M., Shi, X., Wang, Y., Li, W., Yao, W., Lei, Z. and Zhao, R. (2020) In-depth genetic analysis reveals conditioning of polyphenol oxidase activity in wheat grains by *cis* regulation of *TaPPO2A-1* expression level. *Genomics* **112**, 4690-4700.

Liu, H., Ding, Y., Zhou, Y., Jin, W., Xie, K. and Chen, L.L. (2017) CRISPR-P 2.0: An improved CRISPR-Cas9 tool for genome editing in plants. *Mol Plant* **10**, 530-532.

Zhang, S., Zhang, R., Gao, J., Song, G., Li, J., Li, W., Qi, Y., Li, Y. and Li, G. (2021) CRISPR/Cas9-mediated genome editing for wheat grain quality improvement. *Plant Biotechnol J* **19**, 1684-1686.
